# Caught between Two Genes: Accounting for Operonic Gene Structure Improves Prokaryotic RNA Sequencing Quantification

**DOI:** 10.1128/mSystems.01256-20

**Published:** 2021-01-12

**Authors:** Taylor Reiter

**Affiliations:** a Department of Population Health and Reproduction, University of California, Davis, Davis, California, USA

**Keywords:** prokaryote, software, transcriptomics

## Abstract

RNA sequencing (RNA-seq) has matured into a reliable and low-cost assay for transcriptome profiling and has been deployed across a range of systems. The computational tool space for the analysis of RNA-seq data has kept pace with advances in sequencing. Yet tool development has largely centered around the human transcriptome. While eukaryotic and prokaryotic transcriptomes are similar, key differences in transcribed units limit the transfer of wet-lab and computational tools between the two domains. The article by M. Chung, R. S. Adkins, J. S. A. Mattick, K. R. Bradwell, et al. (mSystems 6:e00917-20, 2021, https://doi.org/10.1128/mSystems.00917-20), demonstrates that integrating prokaryote-specific strategies into existing RNA-seq analyses improves read quantification. Unlike in eukaryotes, polycistronic transcripts derived from operons lead to sequencing reads that span multiple neighboring genes. Chung et al. introduce FADU, a software tool that performs a correction for such reads and thereby improves read quantification and biological interpretation of prokaryotic RNA sequencing.

## Abstract (precis)

RNA sequencing (RNA-seq) has matured into a reliable and low-cost assay for transcriptome profiling and has been deployed across a range of systems. The computational tool space for the analysis of RNA-seq data has kept pace with advances in sequencing.

## COMMENTARY

Over the last 15 years, RNA sequencing (RNA-seq) has offered a high-resolution view of the presence and abundance of transcripts at a given time (1, 2). Transcriptome sequencing has revealed the functional elements of genomes and their relationships to cellular environments across a wide range of organisms. Accurate estimation of gene abundances underlies many discoveries from RNA sequencing, including those relying on differential expression analysis and gene coexpression networks (3). Due to the foundational role of accurate transcript estimates, both experimental and computational techniques have been developed to improve the accuracy of read-based quantification methods. While advances have been disproportionately driven in the eukaryotic transcriptome space, many advances improve read quantification across domains of life. For example, due to the presence of similar sequences in a genome such as what occurs with paralogous genes, some transcriptome reads ambiguously map to multiple genes or transcripts at distant locations in the genome. To better assign read counts in these situations, expectation maximization algorithms use counts from unambiguously mapped reads to estimate the true abundance of multimapped reads (4–7). This method improves read quantification in any genome that contains paralogous genes.

Fundamental differences in transcription limit the transfer of innovation in the quantification of transcripts between biological domains. Eukaryotic transcripts contain a single product (monocistronic), while many prokaryotic transcripts contain multiple products (polycistronic, e.g., all genes in an operon). Computational prediction of operon structures is still an active area of research, meaning that laboratory-based techniques like 5′ and 3′ rapid amplification of cDNA ends (RACE) and direct RNA sequencing remain the gold standard for operon prediction but preclude application to the majority of RNA sequencing experiments. Without well-annotated reference transcriptomes that contain polycistronic transcripts, genome-based alignment strategies better capture reads that span genes in an operon. Similar to multimapped reads, reads that span multiple genes create problems in read quantification. However, as a uniquely prokaryotic problem, this problem has received little attention.

With the development of the FADU software tool, Chung and colleagues (8) present a simple and elegant correction for the quantification of reads from prokaryotic transcriptomes that span multiple genes when mapped against a reference genome. The algorithm assigns read counts that are proportional to the length of the overlap between a read and gene, corrected by the length of the gene itself. This method alleviates the undercounting and overcounting of operonic genes and small genes in gene-dense regions by other approaches, thereby improving the accuracy of downstream analyses that rely on gene counts such as differential expression. This concept is illustrated on simulated and real data, demonstrating that proportional correction for reads that span multiple genes impacts the biological interpretation of prokaryotic sequencing data.

This correction is a valuable contribution that is poised for incorporation into general prokaryotic RNA-seq analyses as well as the broader read counting tool space. As a stand-alone tool that operates on BAM alignment files, FADU can be integrated into RNA-seq analysis as an alternative to software like featureCounts or HTSeq. FADU has a small memory and CPU footprint that is permissive for integration into routine RNA-seq analysis pipelines, including for large-scale RNA-seq analyses. Alternatively, many other tools that perform read quantification already provide optional parameters to control behavior around multimapped reads and thus likely contain the infrastructure to support the adoption of this new correction technique (9, 10). Integration of this correction step into other read quantification tools would support widespread adoption. Adoption of this correction will provide relief of systematic biases in the quantification of operonic genes and small genes in gene-dense coding regions, supporting improved biological insights from prokaryotic transcriptomics.

While Chung and colleagues (8) explored their approach in the prokaryotic transcriptome space, correction for reads that map to multiple genes may additionally improve metagenome, metatranscriptome, and single-cell read quantifications. These applications remain to be tested but may improve insights into operonic and gene-dense coding regions in yet-to-be-cultured prokaryotes from diverse environments.
